# *Chlamydia trachomatis* and *Mycoplasma genitalium* Plasma Antibodies in Relation to Epithelial Ovarian Tumors

**DOI:** 10.1155/2011/824627

**Published:** 2011-07-28

**Authors:** Annika Idahl, Eva Lundin, Margaretha Jurstrand, Urban Kumlin, Fredrik Elgh, Nina Ohlson, Ulrika Ottander

**Affiliations:** ^1^Department of Clinical Science/Obstetrics & Gynecology, Umeå University, SE-901 87 Umeå, Sweden; ^2^Department of Medical Biosciences/Pathology, Umeå University, SE-901 87 Umeå, Sweden; ^3^Public Health and Clinical Medicine, Nutritional Research, Umeå University, SE-901 87 Umeå, Sweden; ^4^Clinical Research Centre, Örebro University Hospital, SE-701 85 Örebro, Sweden; ^5^Department of Clinical Microbiology/Virology, Umeå University, SE-901 87 Umeå, Sweden

## Abstract

*Objective*. To assess associations of *Chlamydia trachomatis* and *Mycoplasma genitalium* antibodies with epithelial ovarian tumors. *Methods*. Plasma samples from 291 women, undergoing surgery due to suspected ovarian pathology, were analyzed with respect to *C. trachomatis* IgG and IgA, chlamydial Heat Shock Protein 60-1 (cHSP60-1) IgG and *M. genitalium* IgG antibodies. Women with borderline tumors (*n* = 12), ovarian carcinoma (*n* = 45), or other pelvic malignancies (*n* = 11) were matched to four healthy controls each. *Results*. Overall, there were no associations of antibodies with EOC. However, chlamydial HSP60-1 IgG antibodies were associated with type II ovarian cancer (*P* = .002) in women with plasma samples obtained >1 year prior to diagnosis (*n* = 7). *M. genitalium* IgG antibodies were associated with borderline ovarian tumors (*P* = .01). *Conclusion*. Chlamydial HSP60-1 IgG and *M. genitalium* IgG antibodies are in this study associated with epithelial ovarian tumors in some subsets, which support the hypothesis linking upper-genital tract infections and ovarian tumor development.

## 1. Introduction

Ovarian cancer is the sixth most common cancer among females and the most lethal gynaecologic malignancy with a 16–51% five-year survival rate globally [1]. No firm conclusions are established on the etiology of ovarian cancer, while 5–10% are attributable to genetic predisposition [2, 3]. Microorganisms causing chronic inflammatory disease have become increasingly investigated in the last decade as possible cancer initiators/promoters. *Helicobacter pylori* has been linked to gastric cancer, human papillomavirus to cervical cancer, and Hepatitis B and C virus to liver cancer [4]. *Chlamydia trachomatis* (*C. trachomatis*) is considered a cofactor in cervical cancer and is primarily associated with squamos cell carcinoma of the cervix [5–7]. The role of persistent infection, leading to chronic inflammation, in the pathogenesis of ovarian cancer has received very little consideration, although a history of pelvic inflammatory disease (PID) is in a case-control study correlated to higher risk for ovarian cancer [8]. *C. trachomatis,* the most common cause of PID in the developed world [9–11], is the genital infectious agent that has most often been addressed as a possible tumor initiator/promoter of the ovaries [12–15].

Primary infection with *C. trachomatis,* the most prevalent sexually transmitted bacterium worldwide with an estimated 90 million new cases occurring each year [16], is often asymptomatic and may persist for several months or years [17]. There is evidence that chlamydial bacteria express high levels of chlamydial heat shock protein 60 (cHSP60), suggested to be antiapoptotic, during persistent infections [18, 19], and serum cHSP60 IgG antibodies are in several studies associated with tubal factor infertility (TFI) [20–24]. *Mycoplasma genitalium* (*M. genitalium*) is another sexually transmitted microorganism that has been associated with PID and TFI [25, 26]. Similar to *C. trachomatis* infections, disease caused by *M. genitalium* is often asymptomatic and it often remains undetected [27]. 

It has been proposed that ovarian tumors can be classified into two groups, type I and type II, based on clinical behavior, pathology, molecular genetic differences, and different precursors [28, 29]. Type II tumors constitute most ovarian carcinomas and are rapidly growing, highly aggressive neoplasms that lack well-defined precursor lesions. Many of the type II tumors, are suggested to originate in the tubal fimbria or peritoneum [30], are associated with tubal intraepithelial carcinoma (TIC) and “p53 signatures” and originate in the secretory cells [31]. Both *C. trachomatis *and *M. genitalium *are especially adept at maintaining long-term relationships with their hosts, modulating and evading the immune system, and are shown to cause infections and inflammation of the fallopian tubes [32–34]. Hence, there is a possibility that these infections might play a role in carcinogenesis of the ovary, particularly the type II carcinomas [35].

Given the incomplete biological explanations for the etiology of ovarian cancer and the hypothesis of chronic infection and inflammation as part of ovarian tumor pathogenesis we estimated the associations of plasma *C. trachomatis* IgG, IgA, and cHSP60 IgG and plasma *M. genitalium *IgG antibodies with ovarian tumors.

## 2. Materials and Methods

This study is in part a cohort study to compare the prevalence of antibodies in women with different diagnoses going through surgery due to suspected ovarian pathology, and in part a case-control study to compare the antibody prevalence in women with ovarian tumors with matched controls. The study is approved by the Human Ethics Committee of the Medical Faculty, Umeå University, Sweden.

### 2.1. Study Population

**Figure 1:**
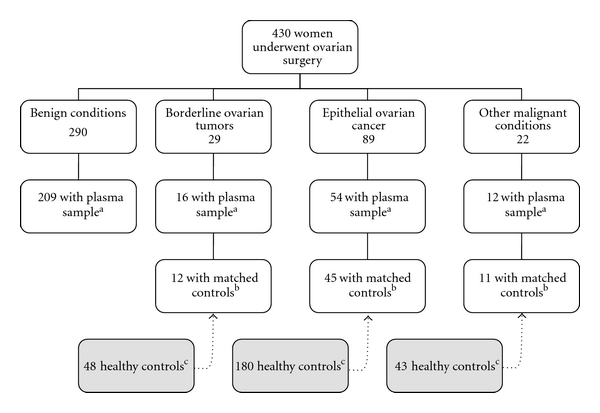
Study cohort originating from women who underwent surgery due to suspected ovarian pathology. ^a^Number of women in the different diagnose groups in whom plasma samples were obtainable. ^b^Number of women for whom 4 matched controls per case were obtainable. ^c^Controls from the NSHDS (Northern Sweden Health and Disease Study) matched with respect to age and time of plasma-sampling.

From 1993 through 2001, 430 women who underwent surgery at the Department of Obstetrics and Gynecology, University Hospital of Umeå, Sweden, due to suspected ovarian pathology were included in the study after oral and written informed consent. The women were mainly from Västerbotten County in northern Sweden. In 238 cases, plasma samples drawn in connection with surgery were available. For another 53 cases, prospective plasma samples (maximum 5.1 years prior to diagnosis) from the Northern Sweden Health and Disease Study (NSHDS) were available and included in the analyses. In total, plasma samples were available for 291 women (Figure 1). In cases of borderline ovarian tumors (BOT), epithelial ovarian cancer (EOC), and other malignancies, control plasma samples from the Medical Biobank of Northern Sweden were matched with respect to age (±1 year) and date of plasma-sampling (±3 months). Age criteria had to be relaxed to ±2 years in 49 out of 271 controls (18%), date of plasma-sampling to ±6 months in 4 controls (1%) and both age and date of plasma-sampling in 4 controls (1%). The control plasma samples were collected as part of the NSHDS, which is a population-based, prospective health survey cohort in Västerbotten County, which has been previously reported in detail [36]. The NSHDS serves the same population as the University Hospital of Umeå regarding ovarian tumor surgery. Control subjects were alive and without a cancer diagnosis (except basalioma) at the time of the diagnosis of the index case. Four matched controls per case were analyzed.

### 2.2. *C. trachomatis* Antibody Analyses

*C. trachomatis*-specific IgG and IgA antibodies, as well as *Chlamydia pneumoniae* (*C. pneumoniae*) IgG antibodies, were determined by the microimmunofluorescence (MIF) test (MRL Diagnostics/Focus, Cypress, CA, USA) specific for serovar D-K, according to the instructions of the manufacturer. Briefly, plasma dilutions at 1/40 (IgG) and 1/16 (IgA) were used. A weak specific immunofluorescence signal in the 1/40 (IgG) and 1/16 (IgA) dilution was reported as positive in 1/20 and 1/8, respectively. Detection of cHSP60 type I (cHSP60-1) IgG antibodies was performed using the commercial cHSP60-1 IgG ELISA technique (Medac, Germany). Protocol and validation criteria of the assay were followed according to the manufacturer's instructions. Briefly, patient plasmas were diluted 1/50 and tested in duplicate. Cut off was defined as the mean optical density (OD) value of the negative control plus 0.350 and results are presented as plus (+) or minus (−). Plasma presenting OD values ±10% of the cut off value were interpreted as indeterminant and were excluded from analysis.

### 2.3. *M. genitalium* Antibody Analysis

*M. genitalium* IgG antibodies were detected using a *M. genitalium* Lipid associated membrane protein-enzyme immuno assay (LAMP-EIA) as previously described [37]. Briefly, 100 *μ*L of plasma samples diluted 1/50 in blocking solution was used. Serum from a patient with a *M. genitalium*—PCR positive result in the urogenital tract specimen was used as a positive control, and pooled sera from blood donors were used as a negative control in each run. The cut off level was set to 0.3 OD and was determined as 3-standard deviations above the negative-control mean.

### 2.4. Clinical Characteristics and Histopathologic Diagnosis

Information on histopathologic diagnosis, according to the World Health Organization classification [38], as well as data on clinical characteristics, was extracted from the medical records. Histopathologic diagnoses were reviewed by a specialist in gynecologic pathology (EL) at the Department of Laboratory Medicine, Clinical Pathology, University Hospital of Umeå. A reclassification according to the proposed hypothesis of a type I and a type II ovarian pathogenetic pathway [29] was done. The type I tumor group consisted of low-grade serous carcinomas and mucinous, endometrioid and clear cell carcinomas. The type II tumor group consisted of ovarian moderate and high-grade serous carcinomas, malignant mixed mesodermal tumors and undifferentiated carcinomas, as well as serous high-grade or undifferentiated peritoneal and fallopian tube cancers.

### 2.5. Statistical Analysis

Statistical analyses were performed using the SPSS software (version 17.0). The pearson Chi-square, and when the expected frequency was <5, Fisher's exact test was used to analyze differences between groups with dichotomous data. Fisher's exact confidence intervals were calculated for the prevalence estimates. Mann-Whitney *U* test was applied to analyze continuous data not normally distributed. A 2-sided *P* value of less than  .05 was considered significant. Odd ratios (OR) and 95% confidence intervals (CI) were calculated using binary logistic regression analysis.

## 3. Results

Clinical characteristics of the women going through ovarian surgery are described in Table 1. Demographic data are not complete, as indicated in the table, particularly among women with benign conditions, but the antibody prevalence was similar in women with or without demographic data. However, women with data on hormone replacement therapy (HRT) use presented *C. trachomatis* IgG (*P* = .023) and cHSP60-1 IgG (*P* = .008) more often than women lacking these data. Women with HRT data were younger (*P* = .006). Women with PID data more often presented *C. trachomatis* IgG (*P* = .046) and cHSP60-1 IgG (*P* = .011) antibodies. *C. trachomatis* IgG antibodies were associated with ever smoking (*P* < .001) and HRT use (*P* = .02), while cHSP60-1 IgG antibodies were associated with ever smoking (*P* = .003) and older age at menarche (*P* = .018). No other associations of antibodies with demographic data were found.

Cases where no plasma samples or controls were available (Figure 1) had the same distribution of diagnoses as cases with matched controls. Reasons for lacking a plasma sample were mostly due to human error when including women about to have surgery. Difficulties in finding controls depend either on high age (>72 years) of the case (*n* = 9) and/or a plasma sample from the first years of the study (1993–1995) (*n* = 5). No differences were found in the prevalence of antibodies between cases with or without matched controls.

The prevalence of *C. pneumoniae* IgG antibodies was 79.8% (95% CI: 76.2%–82.4%) and no covariations of *C. trachomatis* IgG, IgA, or cHSP60-1 IgG antibodies with *C. pneumoniae *IgG antibodies were found. *C. trachomatis* IgG and cHSP60-1 IgG antibodies were associated with a 74% concordance rate.

The prevalence of *C. trachomatis* IgG, cHSP60-1 IgG, and *M. genitalium* IgG antibodies among all women tested, including matched controls, are given in Table 2. 

Notably, there is a significantly higher prevalence of *M. genitalium* IgG antibodies among women with borderline tumors compared with matched controls and women with benign conditions. Three of the four cases positive in *M. genitalium *IgG were also positive in cHSP60-1 IgG. In the subgroup of ovarian cancer cases that had prospective plasma samples collected more than one year prior to diagnosis (*n* = 11), cHSP60-1 IgG antibodies were more prevalent among cases compared with women with benign conditions. The prevalence of plasma *C. trachomatis* IgA antibodies among all cases was 9.6% (95% CI: 6.5%–13.6%) with no association with ovarian tumors.

The antibody prevalences among women with type I tumors, or any of the histological subgroups of EOC, were not raised compared with neither matched controls nor women with benign conditions when plasma samples drawn in connection with diagnosis was analyzed (data not shown). The results of the antibody analyses for the type II ovarian tumors are described in Table 3. In plasma samples drawn in connection with diagnosis in type II tumors, there was no evidently higher prevalence of cHSP60-1 IgG antibodies compared with matched controls or women with benign conditions. However, when prospective plasma samples of the type II tumor group (*n* = 7) were analyzed, the prevalence of cHSP60-1 IgG antibodies was significantly higher than in controls. Six out of seven cases were cHSP60-1 IgG antibody-positive. The clinical characteristics of this subgroup are presented in Table 4. The one woman that was cHSP60-1 IgG antibody-negative had an undifferentiated ovarian cancer. Assigning indeterminant cHSP60-1 IgG antibody results either positive or negative values did not change the main outcomes. 

## 4. Discussion

In this analysis of a cohort of women going through surgery due to suspected ovarian pathology, we found an association of plasma cHSP60-1 IgG antibodies with type II ovarian tumors when prospective plasma samples were analyzed. Additionally, an association of plasma *M. genitalium* IgG antibodies with borderline ovarian tumors was detected. Neither *C. trachomatis *IgG nor IgA was significantly associated with any type of tumors in this cohort. 

Strengths of this study are the population-based selection of case and control subjects with controls matched with respect to age and time of plasma-sampling. Moreover, the cohort was relatively homogeneous, the collection and storage of blood samples was standardized, the measurement of antibodies was performed in a research laboratory with laboratory personnel unaware of case-control status, the *C. trachomatis* IgG and IgA antibody test method is serovariant D-K specific, a standardized cHSP60-1 IgG test method was used, and the *M. genitalium* IgG method has no cross-reactivity to *M. pneumoniae*. Limitations of the study include the small number of subjects with eligible plasma samples and matched controls, making subgroup analysis and analysis of combinations of antibodies difficult. Incomplete data on clinical characteristics reduces the possibility to correct for potential confounders. However, the subgroup of women with type II carcinoma and prospectively drawn plasma samples did not seem to stand out concerning their clinical characteristics. The plasma samples in cases were mostly drawn within a few days prior to surgery. Analyzing plasma samples drawn more than one year prior to diagnosis might have been a more plausible approach considering the result of the subgroup analysis of cases with prospectively drawn plasma samples and previous studies regarding *C. trachomatis* and cervical cancer [39–41].

The association of cHSP60-1 IgG antibodies with ovarian cancer, and the slightly higher prevalence of *C. trachomatis* IgG antibodies, is consistent with the findings of an association of high levels of serum *C. trachomatis* IgG and cHSP60-1 IgG antibodies with ovarian cancer in a population-based case-control study [13]. Wong et al., however, did not find an association of *Chlamydia* serum IgG, IgA, or IgM antibodies with epithelial ovarian cancer [15], maybe because the test method applied was not *C. trachomatis* specific and detected antibodies to all chlamydial species including *C. pneumoniae*. Serum *C. trachomatis* IgG antibodies were not associated with ovarian cancer in the recent study by Ness et al. [14], rather, a protective effect in younger-age groups was found. In that study, serum samples obtained after cancer treatment were analyzed possibly masking an association. Chlamydial HSP60-1 IgG antibodies were the antibodies most clearly associated with ovarian type II tumors in this study. Chlamydial HSP60 expression is found to be increased in prolonged infections with *C. trachomatis* and is suggested to have an antiapoptotic effect. This effect facilitates the survival of the bacteria within the host [18, 19], but at the same time it facilitates the survival of DNA-damaged cells leading to increased risk for cancer initiation [42].

The subgroup of women with prospectively drawn plasma samples displayed larger differences in antibody prevalence between cases and controls than women with plasma samples drawn at the time of diagnosis. Similar to this, concerning cervical cancer, Naucler et al. observed a positive correlation between *C. trachomatis* IgG antibodies and cases observed during followup, but not with cases identified at baseline [39]; cHSP60-1 IgG antibodies have been associated with cervical cancer in cases with long lag time [40]; and *C. trachomatis *DNA in Pap smears taken many years before diagnosis have been associated with cervical cancer [41]. A conceivable explanation for this might be that *C. trachomatis* exerts its effect many years, or decades, before the cancer is overt and that antibodies might decline over time [43–46]. 

Type II tumors are suggested to originate in the secretory cells of the tubal fimbria in a significant percentage of tumors [30, 31, 47, 48]. The secretory cells are the cell type where chlamydial inclusions have been detected in experimental models [49, 50] and the bacteria are known to cause damage to the tubal fimbria. Therefore, it seems plausible that this subgroup was the one associated with cHSP60-1 IgG antibodies. However, the subgroup of women with prospective plasma samples was small, which will raise concern that this result is due to chance. 

Plasma *C. trachomatis* IgA antibodies displayed varying and low prevalence in all groups and were not associated with the finding of ovarian tumors. IgA antibodies, in contrast to IgG and cHSP60-1 IgG, are more likely to reflect an active on-going phase of the infection which might be passed several years before the tumor is overt. 

The association between *M. genitalium* IgG antibodies and borderline ovarian tumors might represent a typeIerror since a Bonferroni correction eliminates the significance of the results. *M. genitalium* is not previously studied in relation to ovarian cancer even though *Mycoplasma* DNA (PCR targeting 15 different species) was found in 59% of ovarian cancer tissues [51]. On the other hand, in a study by Quirk et al., *Mycoplasma* ribosomal DNA (11 different *Mycoplasma* species but not *M. genitalium*) was found not associated with ovarian tumors [52]. In vitro, *M. genitalium* can cause inflammatory changes of the epithelium to the human Fallopian tubes [53]. 

The prevalence of cHSP60-1 IgG antibodies was in this study relatively high compared with the prevalence of *C. trachomatis* IgG antibodies, particularly in the subgroup of women with type II carcinoma. This is different from the findings in most other studies and might reflect differences in laboratory methods. Another explanation is that the *C. trachomatis*, infections that will eventually lead to carcinomatous changes, produce higher amounts of cHSP60 or produces cHSP60 for a more protracted period of time inducing the production of cHSP60-1 IgG antibodies. Alternatively, there is a difference in the host immune system that leads both to the continuous production of cHSP60-1 IgG antibodies and renders the host more susceptible to carcinomatous changes. This might be the case, if for example, an autoimmune cross-reaction to the human HSP60 [54] has a pathological role to play.

In conclusion, we have identified an association of plasma cHSP60-1 IgG antibodies with type II ovarian tumors in the subgroup of women with plasma samples drawn more than one year prior to surgery. This novel finding points toward another possible explanation of the etiology of ovarian neoplasia. *M. genitalium* was weakly associated with borderline ovarian tumors. Defining *C. trachomatis* and *M. genitalium* as disease precursors would have great impact on the importance of preventing, diagnosing, and treating *C. trachomatis *and *M. genitalium* infections to reduce not only fertility related morbidity and chronic pelvic pain, but also ovarian tumor development. However, the results have to be confirmed in larger studies and in studies with prospective plasma samples, and the mechanisms by which the bacteria might initiate or promote tumor development have to be clarified. Nevertheless, this study provides support to the intriguing hypothesis of a link between upper-genital tract infectious agents such as *C. trachomatis,* possibly *M. genitalium,* and ovarian tumor development.

## Figures and Tables

**Table 1:** Clinical characteristics for the women (291) with benign conditions, borderline ovarian tumors, epithelial ovarian cancer, or other pelvic malignancies included in the plasma antibody analyses.

Clinical characteristics	Benign conditions (*n* = 209)	BOT (*n* = 16)	EOC (*n* = 54)	Other pelvic malignancies (*n* = 12)	BOT versus benign, *P* value	EOC versus benign, *P* value
Age, y	52 (18–87)	53 (40–82)	61 (31–78)	58 (40–73)	1.0^b^	.003^b^
Menarche^a^, y	13 (10–16), *n* = 69	13 (11–14), *n* = 4	13 (11–17), *n* = 51	13 (11–16), *n* = 8	.9^b^	.06^b^
Menopause^a^, y	50 (40–57), *n* = 93	50 (44–54), *n* = 7	50 (43–61), *n* = 41	50 (41–55), *n* = 7	1.0^b^	.2^b^
Parity^a^	2 (0–8), *n* = 186	2 (0–4), *n* = 14	2 (0–5), *n* = 54	3 (0–3), *n* = 11	.6^b^	.8^b^
0 children^a^	37 (20%), *n* = 186	1 (7%), *n* = 14	12 (22%)	1 (9%), *n* = 11	.5^c^	.7^d^
≥3 children^a^	49 (26%), *n* = 186	3 (21%), *n* = 14	14 (26%)	6 (54%), *n* = 11	1.0^c^	.9^d^
Oral contraceptive pill use ≥1 year^a^	61 (73%), *n* = 84	4 (80%), *n* = 5	17 (37%), *n* = 46	1 (12%), *n* = 8	1.0^c^	<.001^d^
Past or current HRT use^a^	37 (24%), *n* = 152	3 (27%), *n* = 11	14 (27%), *n* = 52	2 (18%), *n* = 11	.7^c^	.2^d^
Past or current smoking^a^	54 (30%), *n* = 183	6 (50%), *n* = 12	18 (33%), *n* = 54	4 (36%), *n* = 11	.2^c^	.6^d^
History of PID^a^	12 (34%), *n* = 35	1 (14%), *n* = 7	4 (9%), *n* = 43	0 (0%), *n* = 9	.4^c^	.007^d^
BMI^a^	26 (19–41), *n* = 41	24 (21–28), *n* = 5	25 (17–34), *n* = 53	26 (19–30), *n* = 11	.4^b^	.5^b^
Prospective plasma sample >1 year prior to diagnosis	22 (11%)	0	11 (20%)	1 (8%)		
Histology						
Serous	*n* = 52	*n* = 9	*n* = 32	SSPC, *n* = 7		
Mucinous	*n* = 35	*n* = 7	*n* = 5			
Endometrioid	*n* = 26	*n* = 0	*n* = 10	Tubal cancer, *n* = 1 Uterine cancer, *n* = 1 Abdominal cancer, *n* = 1		
	Follicle cyst, *n* = 39 Brenner tumor, *n* = 1 Theca cell tumor, *n* = 1 Fibroma, *n* = 6 Corpus luteum cyst, *n* = 2 Extra uterine pregnancy, *n* = 3 Tubo-ovarian abcess/sacto salpinx, *n* = 6 Endometriosis, *n* = 6 Gastrointestinal inflammation, *n* = 3 Other, *n* = 14 Normal, *n* = 15		Mixed EOC, *n* = 1 Undifferentiated EOC, *n* = 4 Clear cell tumor, *n* = 2	Undifferentiated tubal cancer, *n* = 1 Undifferentiated abdominal cancer, *n* = 1		
Type 1 pathogenetic pathway			19 (35%)	3 (25%)		
Type 2 pathogenetic pathway			35 (65%)	9 (75%)		

Values are presented as medians and range (minimum and maximum values of age, menarche, menopause, parity, and BMI); or number and percentage of patients in each group (0 children, ≥3 children, oral contraceptive pill use ≥1 year, past or current HRT use, past or current smoking, history of PID: prospective plasma samples>1 year prior to diagnosis, type I pathogenetic pathway, and type II pathogenetic pathway).

BOT: borderline ovarian tumor; EOC: epithelial ovarian cancer; HRT: hormone replacement therapy; PID: pelvic inflammatory disease; BMI: body mass index; SSPC: Serous Surface Papillary Carcinoma.

^a^Variable with partial missing data.

^b^Mann-Whitney *U* test.

^c^Fisher's exact test.

^d^Chi-Square test.

**Table 2:** Prevalence of plasma antibodies in women with borderline ovarian tumors, epithelial ovarian cancer, and other pelvic malignancies compared with matched controls and women with benign conditions.

Tumors and antibodies analyzed	Antibody-positive *n* (%)		Cases^a^ versus matched controls^b^,* P* value	Antibody-positive *n* (%)		Cases versus benign conditions, *P* value
BOT	Cases^a^ (*n* = 12)	Matched controls^b^ (*n* = 48)		Cases (*n* = 16)	Benign conditions (*n* = 209)	
* C. trachomatis* IgG	4 (33%)	9 (19%)	.3	5 (31%)	51 (24%)	.5
cHSP60 IgG	4 (33%)	11 (23%)	.5	5 (31%)	48 (24%), *n* = 202^c^	.5
* M. genitalium* IgG	4 (33%)	2 (4%)	.01	4 (25%)	17 (8%)	.049
EOC	Cases^a^ (*n* = 45)	Matched controls^b^ (*n* = 180)		Cases (*n* = 54)	Benign conditions (*n* = 209)	
*C. trachomatis* IgG	9 (20%)	26 (14%)	.4	9 (17%)	51 (24%)	.2
cHSP60 IgG	10 (22%)	38 (22%), *n* = 172^c^	1.0	13 (24%)	48 (24%), *n* = 202^c^	1.0
*M. genitalium* IgG	4 (9%)	6 (3%)	.1	4 (7%)	17 (8%)	1.0
Other pelvic malignancies	Cases^a^ (*n* = 11)	Matched controls^b^ (*n* = 43)		Cases (*n* = 12)	Benign conditions (*n* = 209)	
*C. trachomatis* IgG	1 (9%)	8 (19%)	.7	1 (8%)	51 (24%)	.3
cHSP60 IgG	3 (27%)	10 (23%)	1.0	3 (25%)	48 (24%), *n* = 202^c^	1.0
*M. genitalium* IgG	0	1 (2%)	1.0	0	17 (8%)	.6
EOC with prospective plasma samples^d^	Cases^a^ (*n* = 10)	Matched controls^b^ (*n* = 40)		Cases (*n* = 11)	Benign conditions (*n* = 209)	
*C. trachomatis* IgG	2 (20%)	4 (10%)	.6	2 (18%)	51 (24%)	1.0
cHSP60 IgG	6 (60%)	10 (26%), *n* = 38^c^	.06	7 (64%)	48 (24%), *n* = 202^c^	.008
*M. genitalium* IgG	0 (0%)	1 (2.5%)	1.0	0 (0%)	17 (8%)	1.0

BOT: borderline ovarian tumors; EOC: epithelial ovarian cancer.

^a^Cases with matched controls available.

^b^Four controls per case from the NSHDS (Northern Sweden Health and Disease Study) were matched with respect to age and date of plasma-sampling.

^c^A few subjects had cHSP60 IgG antibody results that were indeterminant and therefore excluded from the statistical analyses. Assigning them extreme values (all positive or negative) did not change the main outcomes.

^d^Epithelial ovarian cancer with plasma samples drawn 1.3 to 5.1 years prior to diagnosis.

Pearson Chi-square, and when the expected frequency was <5, Fisher's exact test was used.

**Table 3:** Prevalence of plasma antibodies in women with malignancies classified as type II tumors (*n* = 44) compared with matched controls and women with benign conditions.

Tumor group and antibodies analyzed	Antibody-positive *n* (%)		Cases^a^ versus matched controls^b^,* P* value	Antibody-positive *n* (%)		Cases versus benign conditions,* P* value	
Type II malignancies	Cases^a^ (*n* = 37)	Matched controls^b^ (*n* = 147)		Cases (*n* = 44)	Benign conditions (*n* = 209)
* C. trachomatis* IgG	8 (22%)	23 (16%)	.4	8 (18%)	51 (24%)	.4	
cHSP60 IgG	10 (27%)	29 (20%)	.4	12 (27%)	48 (24%), *n* = 202^c^	.6	
* M. genitalium* IgG	4 (11%)	5 (3%)	.08	4 (9%)	17 (8%)	.8	
Type II with prospective plasma samples^d^	Cases^a^ (*n* = 7)	Matched controls^b^ (*n* = 28)		Cases (*n* = 7)	Benign conditions with prospective plasma samples (*n* = 22)	Cases versus benign conditions with prospective plasma sample, *P* value	Cases versus all benign conditions, *P* value
* C. trachomatis* IgG	2 (29%)	3 (11%)	.3	2 (29%)	4 (18%)	.6	.7
cHSP60 IgG	6 (86%)	5 (19%), *n* = 26^c^	.002	6 (86%)	6 (30%), *n* = 20^c^	.02	.001
* M. genitalium* IgG	0	0		0	2 (9%)	1.0	1.0

^a^Cases with matched controls available.

^b^Four controls per case from the NSHDS (Northern Sweden Health and Disease Study) were matched with respect to age and date of plasma-sampling.

^c^A few subjects had cHSP60 IgG antibody results that were indeterminant and therefore excluded from the statistical analyses. Assigning them extreme values (all positive or all negative) did not change the main outcomes.

^d^A subgroup of type II malignancies where plasma samples were drawn 1.3 to 5.1 years prior to diagnosis.

The Pearson chi-square was used first,and when the expected frequency was <5, Fisher's exact test was used.

**Table 4:** Clinical characteristics of women with type II tumors and plasma samples drawn 1.3–5.1 years prior to diagnosis.

Clinical characteristics	Type II tumors with prospective plasma samples (*n* = 7)
Age, y	62 (56–69)
Menarche, y	14 (12–16)
Menopause, y	50 (41–60)
Parity	3 (0–5)
0 children	2 (29%)
≥3 children	4 (57%)
Oral contraceptive pill use ≥1 year^a^	2 (33%), *n* = 6
Past or current HRT use	2 (29%)
Past or current smoking	2 (29%)
History of PID	2 (29%)
BMI	26 (20–31)
Histology	
Serous ovarian cancer	4
Mixed epithelial ovarian cancer	1
Undifferentiated ovarian cancer	1
SSPC	1

Values are presented as medians and range (minimum and maximum values of age, menarche, menopause, parity, and BMI); or number and percentage of patients in each group (0 children, ≥3 children, oral contraceptive pill use ≥1 year, past or current HRT use, past or current smoking, history of PID).

HRT: hormone replacement therapy; PID: pelvic inflammatory disease; BMI: body mass index; SSPC: Serous Surface Papillary Carcinoma.

^a^Missing data in one case.
